# The Root Bark of *Morus alba* L. Suppressed the Migration of Human Non-Small-Cell Lung Cancer Cells through Inhibition of Epithelial–Mesenchymal Transition Mediated by STAT3 and Src

**DOI:** 10.3390/ijms20092244

**Published:** 2019-05-07

**Authors:** Tae-Rin Min, Hyun-Ji Park, Moon Nyeo Park, Bonglee Kim, Shin-Hyung Park

**Affiliations:** 1 Department of Pathology, College of Korean Medicine, Dong-eui University, Busan 47227, Korea; rinee@deu.ac.kr (T.-R.M.); 14554@deu.ac.kr (H.-J.P.); 2 Department of Pathology, College of Korean Medicine, Graduate School, Kyung Hee University, Seoul 02447, Korea; mnpark@khu.ac.kr (M.N.P.); bongleekim@khu.ac.kr (B.K.)

**Keywords:** *Morus alba* L., non-small-cell lung cancer, migration, epithelial–mesenchymal transition, STAT3, Src

## Abstract

The root bark of *Morus alba* L. (MA) has been traditionally used for the treatment of various lung diseases in Korea. Although recent research has demonstrated its anticancer effects in several cancer cells, it is still unclear whether MA inhibits the migratory ability of lung cancer cells. The present study investigated the effects of MA on the migration of lung cancer cells and explored the underlying mechanism. Results from a transwell assay and wound-healing assay demonstrated that methylene chloride extracts of MA (MEMA) suppressed the migration and invasion of H1299, H460, and A549 human non-small-cell lung cancer (NSCLC) cells in a concentration-dependent manner. Results from Western blot analyses showed that MEMA reduced the phosphorylation of STAT3 and Src. In addition, MEMA downregulated the expression of epithelial–mesenchymal transition (EMT) marker proteins including Slug, Snail, Vimentin, and N-cadherin, while upregulating the expression of Occludin—a tight-junction protein. The regulation of EMT markers and the decrease of migration by MEMA treatment were reversed once phospho-mimetic STAT3 (Y705D) or Src (Y527F) was transfected into H1299 cells. In conclusions, MEMA inhibited the migratory activity of human NSCLC cells through blocking Src/STAT3-mediated EMT.

## 1. Introduction

Lung cancer is the leading cause of cancer-related deaths among both men and women in the world. It is also the most commonly diagnosed cancer with 2.1 million new lung cancer cases worldwide in 2018 [1]. The major cause of the disease is cigarette smoking, followed by other environmental risk factors including radon, diesel, and ionizing radiation [2]. Most lung cancers are diagnosed at late stages, when they have already local invasion or distal metastases [3]. As 90% of all cancer-related deaths are the result of metastases, rather than of the primary tumors [4], the frequent metastasis of lung cancer contributes to its poor prognosis with an overall five-year survival less than 15% [5]. These facts highlight the need to develop novel therapeutics that effectively suppress the metastasis of lung cancer.

In order to invade and metastasize to other tissues, the epithelial cancer cells acquire and apolar, motile and a mesenchymal-like phenotype, a process called epithelial–mesenchymal transition (EMT). Although the EMT program is essential for normal embryogenesis and repair of wounded tissues, it is also implicated in cancer progression [6,7]. Because mesenchymal cells are highly mobile and invasive, EMT enables carcinoma cells to leave the primary tumor and invade into the local tissue and blood vessels. In addition, EMT confers cancer cells resistance to anoikis upon detachment from the basal lamina [8,9]. Consistently, clinical evidences suggest that EMT correlates with poor prognosis of cancer patients [10,11,12]. Epithelial–mesenchymal transition programs are driven by the activation of several transcription factors including Snail, Slug, and Twist [13,14,15]. Overall, the expressions of cell adhesion molecules such as E-cadherin, Claudins and Occludin are decreased, while mesenchymal markers such as N-cadherin, Vimentin, and Fibronectin are upregulated during EMT [6,7], which results in more transient adhesive properties of cancer cells.

The root bark of *Morus alba* L. (MA) has been traditionally used for the treatment of various lung diseases including cough, hemoptysis, bronchitis, and pulmonary asthma in Korea. More recently, it has been reported that extracts of MA exhibit anti-inflammatory [16], anti-oxidant [17], hypoglycemic [18], and anti-cancer activities [19,20]. However, the effects of MA on the migratory ability of lung cancer cells have not been studied yet. In the current study, we investigated whether MA affects the migration and invasion of human non-small-cell lung cancer (NSCLC) cells and explored the underlying mechanism with focus on EMT regulation.

## 2. Results

### 2.1. Identification of Morusin from MEMA through HPLC Analysis

**Figure 1:**
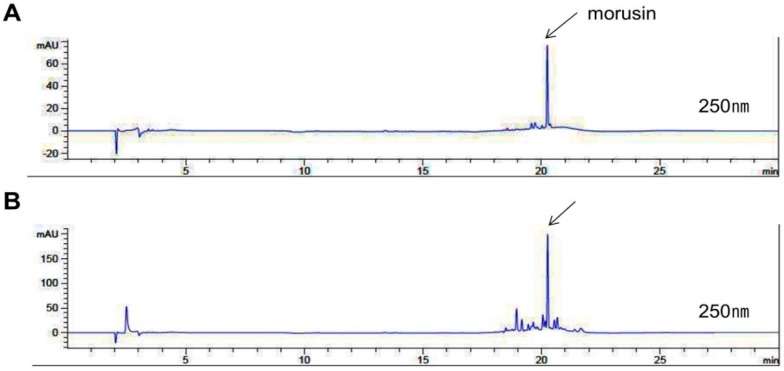
HPLC analysis of standard solution and methylene chloride extracts of *Morus alba* L. (MEMA). Small samples of morusin was separated in parallel with MEMA using HPLC system. Total HPLC-chromatograms of morusin (**A**) and MEMA (**B**) obtained at a UV wavelength of 250 nm. The indicated peak was identified as morusin according to retention time and UV-Vis spectra of standards.

In order to investigate whether a marker component of MA is contained in methylene chloride extracts of MA (MEMA), we performed HPLC analysis. We used morusin as a test compound because morusin exists specifically in Morus species [21,22]. The peak of morusin was detected at a retention time of 20.252 min at an UV wavelength of 250 nm. The chromatogram of MEMA contained various peaks including a peak at a retention time of 20.255 min, indicating that MEMA contained morusin (Figure 1 and Table 1).

### 2.2. MEMA Suppressed the Migration of Human NSCLC Cells

**Figure 2:**
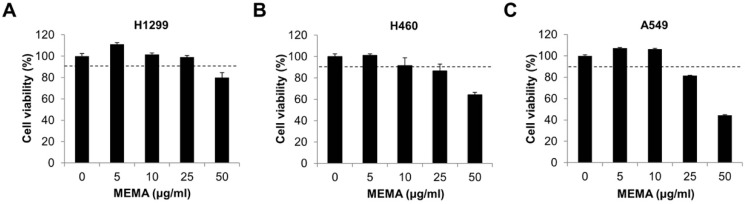
Effects of MEMA on cell viability in human non-small-cell lung cancer (NSCLC) cells. H1299 (**A**), H460 (**B**), and A549 (**C**) human NSCLC cells were treated with MEMA for 48 h. The cell viability was evaluated by MTT assay. Data are expressed as the mean ± SD of three independent experiments. The dotted line indicates the cell viability of 90%.

We performed an MTT assay to determine the concentration that MEMA does not have any cytotoxic effect in human NSCLC cells. The results showed that the cell viability was more than 90% at ≤25 μg/mL of MEMA in H1299 cells, and ≤10 μg/mL of MEMA in H460 and A549 cells, suggesting that MEMA exhibited no obvious cytotoxicity in these ranges (Figure 2A–C). Therefore, we set 25 μg/mL, for H1299 cells, and 10 μg/mL, for H460 and A549 cells, as the maximum concentration of MEMA in further experiments.

**Figure 3:**
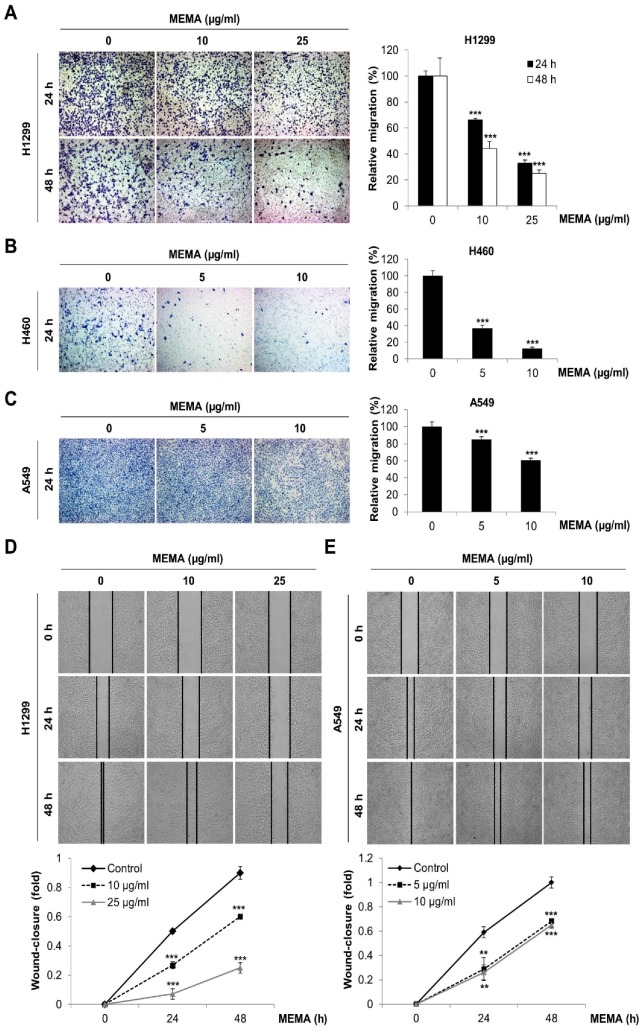
Effects of MEMA on the migration of human NSCLC cells. (**A**–**C**) Transwell migration assay was performed in H1299 (**A**), H460 (**B**), and A549 (**C**) cells. Cells were plated into the gelatin-coated upper chambers of a 24-well format transwell plate and treated with MEMA. Ten percent FBS medium was added in lower chamber as a chemoattractant. After 24 h or 48 h of incubation, the migrated cells were stained and photographed under microscope (100× magnification). The representative fields of three independent experiments are shown (left panel). The relative migration was calculated by counting the number of stained cells (right panel). (**D**,**E**) H1299 (**D**) and A549 (**E**) were seeded onto 6-well plates and the confluent cell layer was scratched with a yellow tip. Then the cells were treated with MEMA in serum-free medium (**D**) or 2% FBS medium (**E**). The wound closure was monitored under microscope during 48 h and photographs were taken at 0 h, 24 h, and 48 h after wound generation (50× magnification). The representative fields of three independent experiments are shown (upper panel), and the wound closure was calculated (lower panel). The data are expressed as the mean ± SD of three independent experiments. Significance was determined by the Student’s *t*-test (** *p* < 0.01, *** *p* < 0.001 versus untreated controls).

In order to evaluate the influence of MEMA on the migration of NSCLC cells, we next conducted transwell migration assay and wound-healing assay. As shown in Figure 3A–C, MEMA treatment markedly reduced the number of migrated cells in NSCLC cells (Figure 3A–C). In addition, MEMA treatment significantly inhibited the wound-healing ability of NSCLC cells in a concentration and time-dependent manner (Figure 3D,E). These results collectively demonstrate that MEMA suppressed the migratory potency of NSCLC cells. 

### 2.3. MEMA Suppressed the Invasion of Human NSCLC Cells

**Figure 4:**
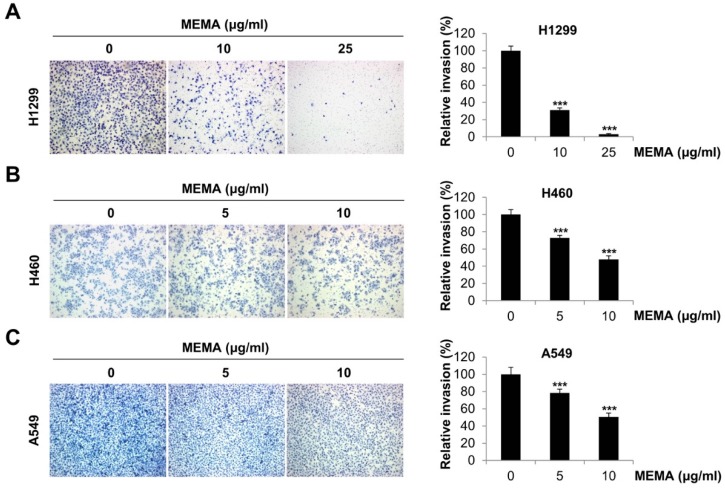
Effects of MEMA on the invasion of human NSCLC cells. Transwell invasion assay was conducted in H1299 (**A**), H460 (**B**), and A549 (**C**) cells. Cells were plated into the matrigel-coated upper chamber of a 24-well format transwell plate and challenged with MEMA. Ten percent FBS medium was used as a chemoattractant. After 24 h of incubation, cells that invaded through the membrane were stained and photographed under microscope (100× magnification). The representative fields of three independent experiments are shown (left panel). The relative invasion was evaluated by counting the number of stained cells (right panel). The data were expressed as the mean ± SD of three independent experiments. Significance was determined by the Student’s *t*-test (*** *p* < 0.001 versus untreated controls).

Invasion to extracellular matrix is one of the critical steps in cancer metastasis [23]. In order to determine the anti-invasive effects of MEMA in NSCLC cells, transwell invasion assay was conducted. As shown in Figure 4, MEMA treatment markedly reduced the number of invaded cells in a concentration-dependent manner, indicating that MEMA inhibited the invasive ability of NSCLC cells (Figure 4A–C). 

To exclude the possibility that proliferating cells are misunderstood as migrating or invading cells, we performed a wound-healing assay and transwell assay at early time points. The wound-healing ability of NSCLC cells was suppressed by 12 h treatment of MEMA (Figure S1A). Results from transwell assay also exhibited that MEMA reduced the number of migrated or invaded cells at 12 h post-treatment of MEMA in NSCLC cells (Figure S1B). These results collectively demonstrate that the inhibitory effects of MEMA on the cell migration and invasion were not caused by suppression of the cell proliferation.

### 2.4. MEMA Suppressed the Activity of STAT3 and Src in Human NSCLC Cells

**Figure 5:**
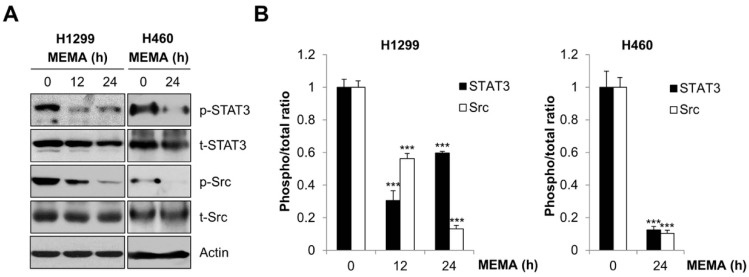
Effects of MEMA on the activity of Src and STAT3 in human NSCLC cells. (**A**) H1299 and H460 cells were treated with 25 or 10 μg/mL of MEMA, respectively, for indicated time periods. The expression levels of the indicated proteins were assessed by Western blot analysis. Actin was used as a loading control. (**B**) The ratio of p-STAT3/t-STAT3 and p-Src/t-Src was calculated using Image J software after normalization with actin. The data were expressed as the mean ± SD of three independent experiments. Significance was determined by the Student’s *t*-test (*** *p* < 0.001 versus untreated controls).

We next explored the molecular mechanism through which MEMA regulated the migration and invasion of NSCLC cells. It is well recognized that signal transducer and activator of transcription 3 (STAT3) and Src are implicated in the promotion of cellular motility and invasion critical for cancer metastasis [23,24]. Therefore, we investigated whether MEMA regulates the activity of STAT3 and Src. As we expected, the phosphorylation of STAT3 and Src was commonly decreased by MEMA in both H1299 and H460 cells, while the expression of corresponding total proteins remained unchanged (Figure 5A,B). Given that STAT3 plays an opposing role to STAT1 which promotes antitumor effects and immunosurveillance [25,26], we next investigated the expression of p-STAT1 after MEMA treatment. As shown in Figure S2, the phosphorylation level of STAT1 was enhanced by MEMA in both H1299 and H460 cells, supporting the idea that STAT1 and STAT3 mutually inhibit the activation of each other (Figure S2). Taken together, these results suggest that regulation of STAT3 and Src might be involved in the inhibitory effects of MEMA on the migration of NSCLC cells.

### 2.5. MEMA Suppressed the Cellular Migration of Human NSCLC Cells through Regulation of EMT Mediated by STAT3 and Src

**Figure 6:**
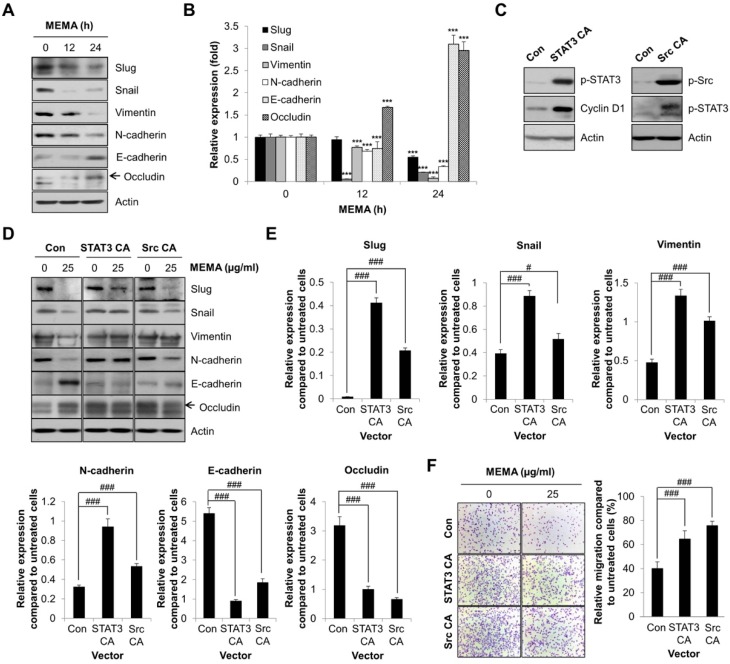
Influence of Src and STAT3 on the regulation of EMT and migration in H1299 cells. (**A**,**B**) H1299 cells were treated with MEMA (25 μg/mL) for 12 or 24 h. The expression levels of EMT marker proteins were assessed by Western blot analysis. (**A**) The representative gel images are shown. (**B**) The relative expressions of the indicated proteins were calculated using Image J software. Actin was used for normalization. (**C**–**E**) H1299 cells were transfected with STAT3 CA or Src CA. (**C**) At 48 h post-transfection, the expression levels of the indicated proteins were assessed by Western blot analysis. (**D**,**E**) At 24 h post-transfection, cells were treated with MEMA (25 μg/mL) for 24 h. The expression levels of EMT marker proteins were assessed by Western blot analysis. (**D**) The representative gel images are shown. (**E**) The relative expressions of the indicated proteins were calculated using Image J software. Actin was used for normalization. (**F**) H1299 cells transfected with either STAT3 CA or Src CA were plated into the gelatin-coated upper chambers of transwell plate and treated with MEMA (25 μg/mL). 10% FBS medium was used as a chemoattractant. After 24 h of incubation, the migrated cells were stained and photographed under microscope (100× magnification). The relative migration of MEMA-treated cells compared to that of untreated cells was calculated by counting the number of stained cells. The data are expressed as the mean ± S.D. of three independent experiments. Significance was determined by the Student’s *t*-test (*** *p* < 0.001 vs. untreated controls; ^#^ *p* < 0.05, ^###^ *p* < 0.001 versus respective control). Con, control vector; STAT3 CA, constitutively active STAT3 (Y705D) vector; Src CA, constitutively active Src (Y527F) vector.

EMT is an emerging paradigm to explain the progression of indolent carcinoma in situ to aggressive metastatic disease [27]. Thus, we investigated the influence of MEMA on the expression of EMT-related proteins. First, we examined the expression of Slug and Snail, most notable transcription factors involved in EMT program. The results showed that Slug and Snail were gradually downregulated by MEMA (Figure 6A,B). Next, we investigated the expression of N-cadherin and Vimentin. During the EMT program, epithelial markers E-cadherin and integrins are replaced by mesenchymal markers Vimentin and N-cadherin, respectively [6]. Our results consistently showed that Vimentin and N-cadherin were significantly down-regulated by MEMA (Figure 6A,B). Finally, MEMA treatment enhanced the expression of Occludin, a tight-junction protein, and E-cadherin (Figure 6A,B). Collectively, these results obviously indicate that MEMA inhibited EMT in NSCLC cells.

Both STAT3 and Src are commonly reported to regulate the initiation and resolution of EMT programs in malignant cells [27,28]. To verify whether the inhibition of STAT3 and Src contributes to the regulation of EMT marker proteins following MEMA treatment, we transfected NSCLC cells with either constitutively active STAT3 (STAT3 CA) or constitutively active Src (Src CA). First, the phosphorylation level of STAT3 or Src was significantly increased by transfection of STAT3 CA or Src CA, indicating that the transfections were successful (Figure 6C). In addition, the expression of cyclin D1, a target protein of STAT3, and the phosphorylation of STAT3, a well-known downstream target of Src, was commonly increased by overexpression of STAT3 CA or Src CA, respectively, indicating that STAT3 and Src were constitutively activated (Figure 6C). We next investigated the expression of EMT markers in H1299 cells transfected with STAT3 CA or Src CA after MEMA treatment. The expression of Slug, Snail, Vimentin, and N-cadherin, commonly decreased by MEMA treatment, were partially or completely recovered by transfection of STAT3 CA or Src CA in H1299 cells (Figure 6D,E). On the other hand, MEMA-induced upregulation of E-cadherin and Occludin was markedly reduced by overexpression of STAT3 CA or Src CA (Figure 6D,E). Similar results were obtained when the same experiments were conducted in H460 cells (Figure S3A–C). These results clearly demonstrate that MEMA regulated the expression of EMT-related proteins by suppressing the activity of STAT3 and Src.

Finally, we confirmed whether inhibition of STAT3 and Src is a pivotal mechanism through which MEMA suppresses the migration of human NSCLC cells. The overexpression of either STAT3 CA or Src CA itself enhanced the cell migration in NSCLC cells (Figure 6E and Figure S3D). In control vector-transfected H1299 cells, MEMA reduced the migration of cancer cells by 40.1% compared to the untreated cells. However, transfection of STAT3 CA or Src CA in H1299 cells increased the migratory ability of cancer cells following MEMA treatment by 64.47% and 75.56%, respectively, compared with the untreated cells (Figure 6F). Similar results were obtained when the same experiments were conducted using H460 cells (Figure S3D). Taken together, these data obviously suggest that MEMA suppressed the migration of human NSCLC cells through inhibition of EMT mediated by STAT3 and Src.

## 3. Discussion

In the current study, we investigated the inhibitory effects of MEMA on the cellular migration and invasion of human NSCLC cells, and explored the underlying mechanism. The novelty of this study is as follows: (i) This is the first study that demonstrated the anti-migration effect of the root bark of MA in cancer cells. Even though there have been a variety of studies suggesting the anti-cancer effects of MA, most of them focused on the apoptotic effect of MA; (ii) We, for the first time, showed that MA regulated EMT marker proteins; (iii) There was no previous study reporting the critical role of STAT3 and Src in mediating the anti-cancer effects of MA. We also demonstrated the association of STAT3 and Src with the regulation of EMT. 

Src plays a definitive role in tumor metastasis by regulating cell migration, adhesion, and invasion [24]. Recent studies have identified that Src is implicated in EMT of cancer cells even though the molecular mechanism has not been fully elucidated. High expression of Src in cancer contributes to the disassembly of adherent junctions and tight junctions, which is associated with upregulation of mesenchymal markers as well as downregulation of epithelial markers [28,29]. STAT3 also plays a key role in EMT as it regulates the activity of the master EMT transcription factors such as Snail, Slug, and Twist [27,30]. Consistently, our data demonstrated that the expression change of EMT markers following MEMA treatment was partially or completely reversed by overexpression of Src CA or STAT3 CA in NSCLC cells, suggesting that MEMA inhibited EMT through regulation of Src and STAT3 activity.

However, it is still possible that regulation of EMT is not the sole mechanism of the anti-migration effects of MEMA based on the following points: (i) Although the expression of EMT marker proteins, up-regulated or down-regulated by MEMA, was almost recovered by overexpression of STAT3 CA, the migratory ability of NSCLC cells was not completely compensated by transfection of STAT3 CA (Figure 6). (ii) Rather, overexpression of Src CA, which did not completely compensate the expression changes of EMT markers following MEMA treatment, exhibited better effects in recovery of migratory potency of NSCLC cells (Figure 6). This might be derived from the concept that morphologic EMT can be uncoupled from molecular EMT. Recent studies demonstrated that mesenchymal motility can occur without regulation of EMT markers [31]. Otherwise, it can be due to the extensive role of Src in regulating cellular migration not only through EMT, but also through other mechanisms such as regulation of cytoskeleton, dissociation of cell–cell adherent junctions, and stabilization of focal adhesion complexes [24].

Previous studies demonstrated that H1299 is a mesenchymal cell line [32,33]. H460 and A549 cells also possess more mesenchymal traits than epithelial traits [34]. As partial EMT is more associated with aggressive cancer progression than complete EMT [35], whether MEMA completely blocks EMT to produce an epithelial cell phenotype or just partially suppresses EMT to generate hybrid E/M phenotype can be an important issue. Because MEMA regulated not only mesenchymal markers but also epithelial markers in protein levels, we suggest that MEMA stimulated a conversion of mesenchymal cell lines into epithelial cell phenotype, not into hybrid E/M phenotype. The current study has not determined how Src and STAT3 crosstalk with each other during the pharmacological action of MEMA. Generally, activation of STAT3 relies on the phosphorylation of a conserved tyrosine residue (Y705) by upstream tyrosine kinases, including receptor tyrosine kinases (RTKs), Janus kinase (JAK), as well as cytoplasmic tyrosine kinases, such as Src. In addition, several RTKs including epithelial growth factor receptor (EGFR) require Src to activate STAT3 [36,37]. Based on these previous studies, we carefully propose that suppression of Src by MEMA might have augmented the inhibitory effect of MEMA on STAT3 activity. Strong phosphorylation of STAT3 by transfection of Src CA in H1299 cells supports our hypothesis (Figure 6C). On the contrary, the phosphorylation of Src was not altered by overexpression of STAT3 CA (data not shown). Identifying the upstream target of Src and STAT3 and determining the crosstalk between Src and STAT3 during MEMA-suppressed cell migration would be our next goal to explore. 

The active compound of MEMA which exhibits anti-migration effects in cancer cells has not been identified in this study. Notably, it is reported that morusin suppressed cancer cell migration and regulated EMT markers by suppression of STAT3 [38]. Ursolic acid is another candidate as it inhibited cancer metastasis by targeting STAT3 or Src [39,40]. Whether the anti-migration effects of MEMA derived from morusin, or from ursolic acid, or from another unknown constituent, or from a synergistic effect among various constituents should be further determined. 

In conclusion, our findings provide a novel insight into the mechanism through which the root bark of MA suppressed the migratory and invasive potency of human NSCLC cells. We demonstrate that STAT3 and Src-mediated EMT regulation played a pivotal role during the pharmacological action of MEMA. Even though further studies are needed to evaluate the effectiveness of MEMA in additional preclinical and clinical settings, we cautiously suggest that MEMA could be an alternative therapeutic option for advanced NSCLC.

## 4. Materials and Methods

### 4.1. Preparation of MEMA

Dried root barks of MA were bought from Nuri Herb Co., Ltd. (Youngcheon, Korea). MA (100 g) was chopped and extracted repeatedly with 1.5 L of methylene chloride for 24 h at room temperature with shaking and occasional sonication. The extract was filtered and concentrated with a vacuum rotary evaporator under reduced pressure and lyophilized. The powder was dissolved as a stock solution at 50 mg/mL in DMSO and stored at −80 °C. 

### 4.2. HPLC Analysis

We conducted chromatographic analysis using Agilent series 1290 system (Agilent Technologies, Palo Alto, CA, USA). The standard morusin (ChemFaces, Wuhan, China) was dissolved in 30% acetonitrile in water. The gradient elution solvents were 0.1% phosphoric acid in water (solvent A) and 50% acetonitrile in water (solvent B) at a flow rate of 0.8 mL/min. The mobile condition performed was as follows: 5% (B) for 0 min, 5% (B) for 5 min, 25% (B) for 6 min, 35% (B) for 13 min, 75% (B) for 15 min, 95% (B) for 17 min, 5% (B) for 21 min, 3% (B) for 22 min. The chromatographic separation was performed at 30 °C and the used wavelength for detection of morusin was 250 nm. 

### 4.3. Cell Culture

Human NSCLC cell lines H1299, H460, and A549 were purchased from the American Type Culture Collection (ATCC; Manassas, VA, USA). Cells were grown in RPMI 1640 (WelGENE, Daegu, Korea) supplemented with 10% fetal bovine serum (FBS, WelGENE) and 1% Penicillin–Streptomycin Solution (WelGENE) at 37 °C in a humidified incubator under 5% CO_2_.

### 4.4. MTT Assay

Three × 10^3^ cells were seeded onto 96-well plates and treated with MEMA. The MTT (3-(4,5-dimethylthiazol-2-yl)-2,5-diphenyltetrazolium bromide; Duchefa, Haarlem, The Netherlands) solution was then added to the media at final concentration of 0.4 mg/mL and incubated for 2 h at 37 °C. The supernatant was removed and 100 μL of DMSO was added to dissolve the formazan. The absorbance of each well was measured at 540 nm using a microplate reader (SpectraMax M3; Molecular Devices, San Jose, CA, USA).

### 4.5. Transwell Assay

For transwell migration assay, 24-well transwell with 8.0 µm pore size (Corning, NY, USA) was used. The outer membrane of insert was coated with 0.1% gelatin (Sciencell, Carlsbad, CA, USA). Two × 10^4^ cells in serum-free medium containing MEMA were seeded onto the upper chamber with the addition of 10% FBS medium as chemoattractant in the lower chamber. After incubation for 24 h or 48 h, the migrated cells were stained with hematoxylin, and counted using a microscope (Carl Zeiss, Oberkochen, Germany). The invasion assay was performed in the same manner with transwell migration assay except that the inner membrane of upper well was coated with 300 μg/mL of Matrigel (BD Bioscience, San Jose, CA, USA). 

### 4.6. Wound-Healing Assay

Eight × 10^5^ cells were seeded in 6-well plates. When cells were 100% confluent, a 200-µL pipette tip was used to make a vertical wound down through the cell monolayer. Then the culture media was replaced by fresh serum-free media (for H1299) or 2% FBS media (for A549) containing MEMA. The wound closure was monitored for 48 h under the microscope (DMI8; Leica, Wetzlar, Germany). The wound closure was calculated using Leica Application suit (Las V4.8) software. 

### 4.7. Transfection

Constitutively active STAT3 plasmid (pExpress-STAT3Y705D) was a gift from Professor Ho-Young Lee (Seoul National University, Korea), and constitutively active Src plasmid (pcSrc527) was purchased from Addgene (#17675). Three × 10^5^ cells were seeded in 6-well plate and transfected with pExpress-STAT3Y705D or pcSrc527 using Lipofectamine2000 (Invitrogen, Carlsbad, CA, USA) according to the manufacturer’s instruction. 

### 4.8. Western Blot Analysis

Cells were lysed with RIPA buffer (Thermo Scientific, Schaumburg, IL, USA) supplemented with a protease inhibitor cocktail (Thermo Scientific) and phosphatase inhibitors (1 mM Na_3_VO_4_ and 100 mM NaF). Equivalent amounts of protein were separated by sodium dodecyl sulfate (SDS)-polyacrylamide gels and transferred to polyvinylidene fluoride (PVDF) membranes (Millipore, Bedford, MA, USA). The membranes were probed with the specific primary and secondary antibodies. The protein–antibody complexes were detected using Super Signal West Pico Chemiluminescent Substrate (Thermo Scientific). Primary antibodies against phospho-STAT3 (Y705), phospho-Src (Y527), STAT3, Src, and Snail were purchased from Cell Signaling Technology (Beverly, MA, USA), and the other primary antibodies were purchased from Santa Cruz Biotechnology (Santa Cruz, CA, USA). Anti-rabbit secondary antibody and anti-mouse secondary antibody were purchased from Enzo Life Sciences (Farmingdale, NY, USA) and Bethyl Laboratories (Montgomery, TX, USA), respectively.

### 4.9. Statistical Analysis

Each result was expressed as the mean ± SD of data obtained from triplicate experiments. A statistical analysis was performed by a paired Student’s *t*-test. Differences at *p* < 0.05 were considered statistically significant.

## Figures and Tables

**Table 1:** Comparison of retention time between MEMA and standard morusin by HPLC analysis.

	Inter-Day		Intraday	
	RT ^1^ (min)	RSD ^2^ (%)	RT ^1^ (min)	RSD ^2^ (%)
Morusin	20.252	2.3094 × 10^−3^	20.254	0
MEMA	20.255	7.0711 × 10^−4^	20.254	1.1547 × 10^−3^

^1^ RT: retention time, ^2^ RSDs: relative standard deviations.

## Supplementary Materials

Supplementary materials can be found at https://www.mdpi.com/1422-0067/20/9/2244/s1.

## Abbreviations

MA	*Morus alba* L.
MEMA	Methylene chloride extract of *Morus alba* L.
EMT	Epithelial–mesenchymal transition
HPLC	High-performance liquid chromatography
MTT	3-(4,5-dimethylthiazol-2-yl)-2,5-diphenyltetrazolium bromide (a tetrazole)
RT-PCR	Reverse transcriptase-polymerase chain reaction
STAT3	Signal transducer and activator of transcription 3
