# Supplementary material for: The Root Bark of Morus alba L. Suppressed the Migration of Human Non-Small-Cell Lung Cancer Cells through Inhibition of Epithelial–Mesenchymal Transition Mediated by STAT3 and Src
Source: Int J Mol Sci. 2019 May 7;20(9):2244. doi: 10.3390/ijms20092244 (PMC6539721; doi:10.3390/ijms20092244)
Supplement: Supplementary file 1 [file ijms-20-02244-s001.pdf]

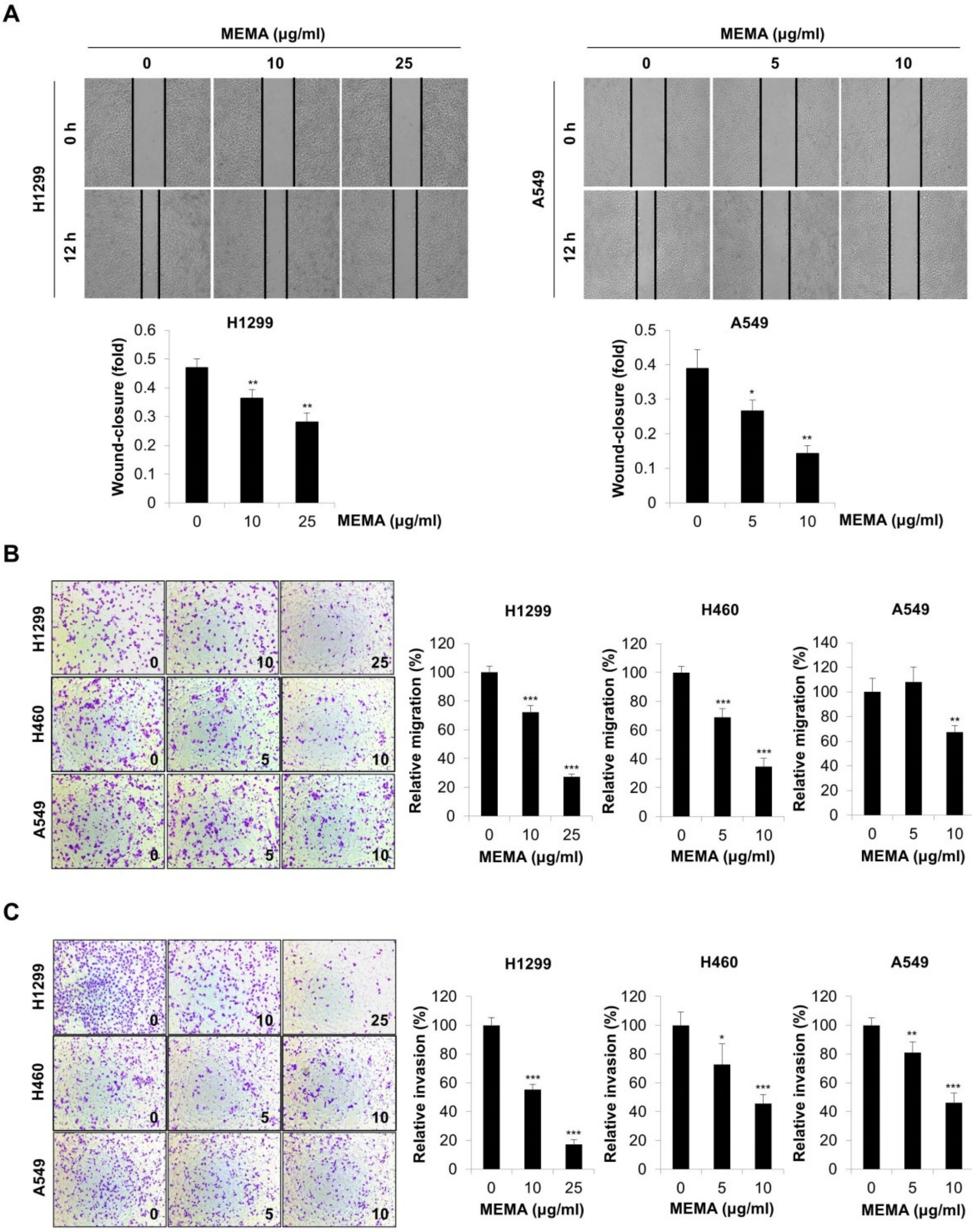

Supplementary Figure 1. Effects of MEMA on the migration and invasion of human NSCLC cells at early time point. (A) H1299 (left) and A549 (right) cells were seeded onto 6-well plates and the confluent cell layer was scratched with yellow tip. Then the cells were treated with MEMA in serum free medium for H1299 cells or in 2% FBS medium for A549 cells. The wound closure was monitored at 0 h and 12 h after wound generation (×50 magnification). The representative fields of three independent experiments are shown (upper panel), and the wound closure was calculated (lower panel). (B and C) Transwell migration assay (B) and transwell invasion assay (C) were performed in H1299, H460, and A549 cells. Cells were plated into the gelatin-coated (B) or Matrigel-coated upper chambers of 24-well format transwell plate and treated with MEMA. 10% FBS medium was added in lower chamber as a chemoattractant. After 12 h of incubation, the migrated or invaded cells were stained and photographed under microscope (×100 magnification). The representative fields of three

independent experiments are shown (left panel). The relative migration was calculated by counting the number of stained cells (right panel). The data are expressed as the mean ± S.D. of three independent experiments. Significance was determined by the Student's t-test (*$P < 0.05$, **$P < 0.01$, *** $P < 0.001$ vs. untreated controls).

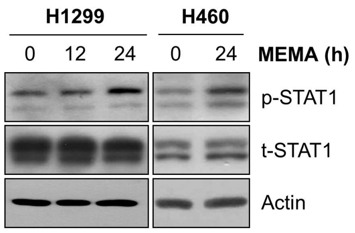

Supplementary Figure 2. Effects of MEMA on the phosphorylation of STAT1. H1299 (left) and H460 (right) cells were treated with 25 μg/ml or 10 μg/ml of MEMA for indicated time periods. The expression levels of p-STAT1 and t-STAT1 were assessed by western blot analysis. Actin was used as a loading control.

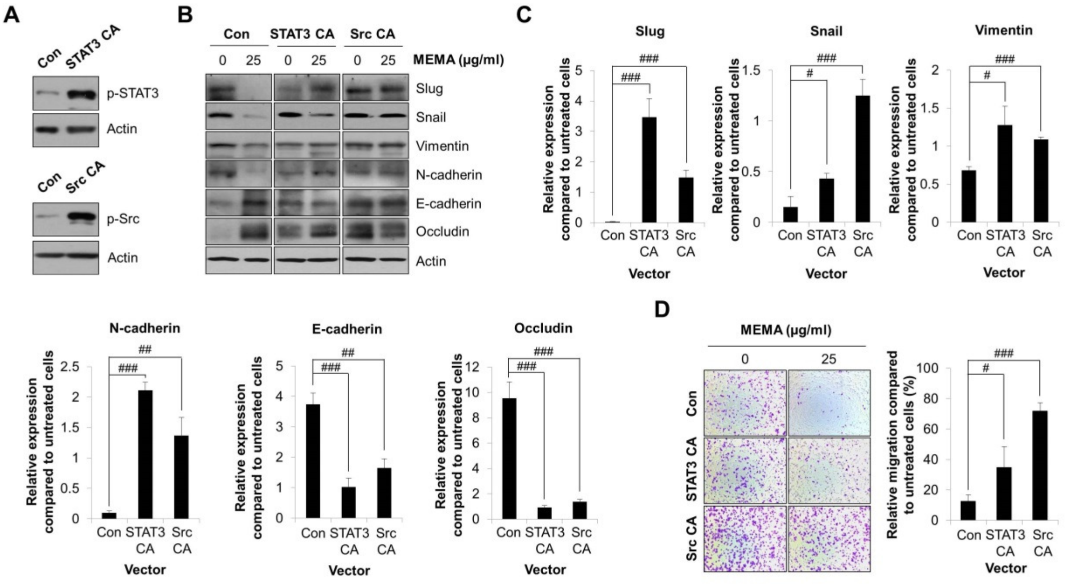

Supplementary Figure 3. Influence of Src and STAT3 on the regulation of EMT and migration in H460 cells. (A) H460 cells were transfected with either STAT3 CA or Src CA. At 48 h post-transfection, the expression levels of the indicated proteins were assessed by western blot analysis. (B,C) H460 cells were transfected with either STAT3 CA or Src CA. At 24 h post-transfection, cells were treated with MEMA (10 µg/ml) for 24 h. (B) The expression levels of EMT marker proteins were assessed by western blot analysis. (C) The relative expressions of the indicated proteins were calculated using Image J software. Actin was used for normalization. (D) H460 cells transfected with either STAT3 CA or Src CA were plated into the gelatin-coated upper chambers of transwell plate and treated with MEMA (10 µg/ml). 10% FBS medium was used as a chemoattractant. After 24 h of incubation, the migrated cells were stained and photographed under microscope (×100 magnification). The relative migration of MEMA-treated cells compared to that of untreated cells was calculated by counting the number of stained cells. The data are expressed as the mean ± S.D. of three independent experiments. Significance was determined by the Student's t-test (# $P < 0.05$, ## $P < 0.01$, ### $P < 0.001$ vs. respective control). Con, control vector; STAT3 CA, constitutively active STAT3 (Y705D) vector; Src CA, constitutively active Src (Y527F) vector.
